# Whole-Eye Radiation for the Local Control of Choroidal Lymphoma in Primary Central Nervous System Lymphoma: A 14-Year Case Study

**DOI:** 10.7759/cureus.85680

**Published:** 2025-06-10

**Authors:** Toshihiko Matsuo, Tomofumi Yano, Kotaro Yoshio, Takehiro Tanaka, Hirotake Nishimura, Ken-ichi Matsuoka

**Affiliations:** 1 Graduate School of Interdisciplinary Science and Engineering in Health Systems, Okayama University, Okayama, JPN; 2 Department of Ophthalmology, Okayama University Hospital, Okayama, JPN; 3 Department of Internal Medicine, Okayama Rosai Hospital, Okayama, JPN; 4 Department of Radiology, Graduate School of Medicine, Dentistry, and Pharmaceutical Sciences, Okayama University, Okayama, JPN; 5 Department of Pathology, Graduate School of Medicine, Dentistry, and Pharmaceutical Sciences, Okayama University, Okayama, JPN; 6 Department of Pathology, Kawasaki Medical School, Kurashiki, JPN; 7 Department of Hematology and Oncology, Graduate School of Medicine, Dentistry, and Pharmaceutical Sciences, Okayama University, Okayama, JPN; 8 Department of Hematology, Endocrinology, and Metabolism, Institute of Biomedical Sciences, Tokushima University Graduate School, Tokushima, JPN

**Keywords:** brain biopsy, bruton tyrosine kinase (btk) inhibitor, chemotherapy, diffuse large b-cell lymphoma, fluorodeoxyglucose positron emission tomography, primary central nervous system lymphoma, primary intraocular (vitreoretinal) lymphoma, radiation therapy (radiotherapy), tirabrutinib, whole-eye radiation

## Abstract

Involved-site radiation therapy is effective for curative and palliative treatments of cancers, including lymphoma. This case study describes the use of whole-eye radiation for primary intraocular lymphoma occurring during primary central nervous system lymphoma. The patient, a 68-year-old man, developed personality changes and apathy two weeks after cataract surgery combined with vitrectomy for vitreous opacity in the left eye. Magnetic resonance imaging revealed a mass lesion in the left frontal lobe, and biopsy by craniotomy confirmed diffuse large B-cell lymphoma. He underwent chemotherapy using rituximab combined with high-dose methotrexate and high-dose cytarabine in association with intrathecal methotrexate and cytarabine injections, leading to complete remission. At age 75, he noticed forgetfulness, and fluorodeoxyglucose positron emission tomography and magnetic resonance imaging revealed a relapse of lymphoma in the splenium of the corpus callosum. He underwent chemotherapy using rituximab combined with high-dose methotrexate, followed by monthly rituximab monotherapy for one year and then rituximab monotherapy every two months for one year. He maintained complete remission with no treatment until age 78, when he developed subretinal choroidal lesions in the left eye and underwent whole-eye radiation at 40 Gy. One year later, he developed subretinal choroidal lesions in the right eye and underwent whole-eye radiation at 40 Gy. At age 81, he had lower limb weakness with disorientation. Magnetic resonance imaging showed a relapse of lymphoma in the right frontal to temporal lobe. The brain lesions showed a marked response to four weeks of oral tirabrutinib as a salvage therapy, but the lesions regrew, and the patient died seven months later. Throughout the treatment, he maintained a visual acuity of 0.7 (decimal scale) in both eyes. In conclusion, whole-eye radiation should be considered as a treatment option for the local control of active intraocular lymphoma, especially choroidal lesions, for patients with primary central nervous system lymphoma with no active brain lesions and without systemic treatment.

## Introduction

Surgical resection is a mainstay treatment for cancers without distant metastases. For surgically unresectable cancers and distant metastases, radiation therapy remains an important treatment option, even in the era of immunotherapy and chemotherapy with molecular-targeted drugs. In addition to curative treatment, radiotherapy can also be used to provide palliative care and pain relief, especially in advanced cancers [1,2]. Lymphoma is the uncontrolled proliferation of lymphocytes and is classified into two types, B-cell and NK/T-cell, with additional subclasses based on the origin of the lymphoma cells. Treatment is chosen according to these classifications. Lymphoma cells are highly sensitive to radiation, making both curative and palliative radiotherapy useful treatment options for these patients.

Primary intraocular lymphoma, or vitreoretinal lymphoma in the updated terminology, is a part of primary central nervous system lymphoma as a rare entity of diffuse large B-cell lymphoma [3]. Lymphoma cells in this entity have a homing tendency to the nervous system and, thus, are prone to develop intraocular lesions since the retina is also a part of the central nervous system. The intraocular lesions manifest as vitreous opacity or retinal infiltration or choroidal infiltration mainly in the subretinal pigment epithelial space or their combination [4-8]. Intraocular lymphoma may precede, accompany, or follow primary central nervous system lymphoma. In this case study, we describe an elderly man with choroidal lymphoma lesions in both eyes who underwent whole-eye radiation during the period of complete remission of brain lesions, and, hence, no treatment, after he had received two rounds of chemotherapy for the initial lesion and a relapse of primary central nervous system lymphoma.

## Case presentation

**Figure 1:**
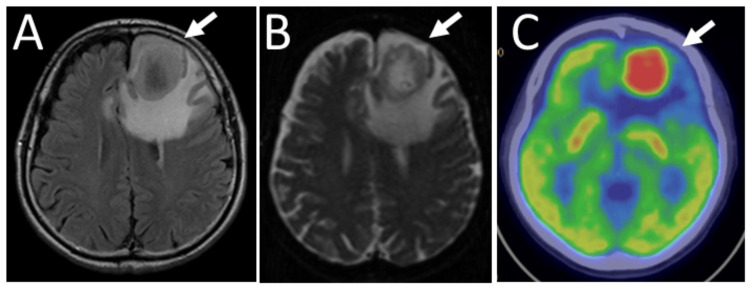
Magnetic resonance imaging and positron emission tomography at age 68 Magnetic resonance imaging of a patient at age 68 showing a large left frontal lobe mass with central necrosis (arrow) and midline shift on T2-weighted FLAIR image (A); diffusion-weighted image (B); high uptake (arrow) in fluorodeoxyglucose positron emission tomography (C) FLAIR: fluid-attenuated inversion recovery

**Figure 2:**
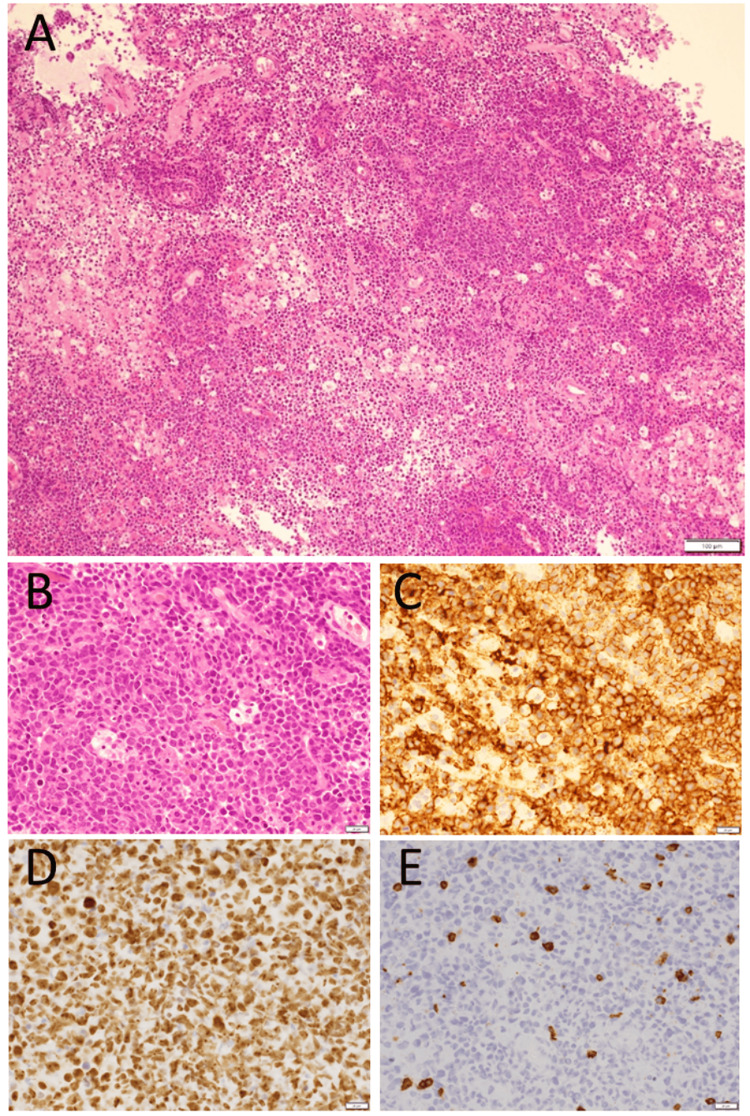
Brain biopsy pathology Images of the patient's brain biopsy by craniotomy at age 68 showing large anomalous cells (A, B) in hematoxylin-eosin stain, positive for CD20 (C) and Ki-67 (D) and negative for CD3 (E). Bar: 100 µm in (A) and 20 µm in (B-E)

**Figure 3:**
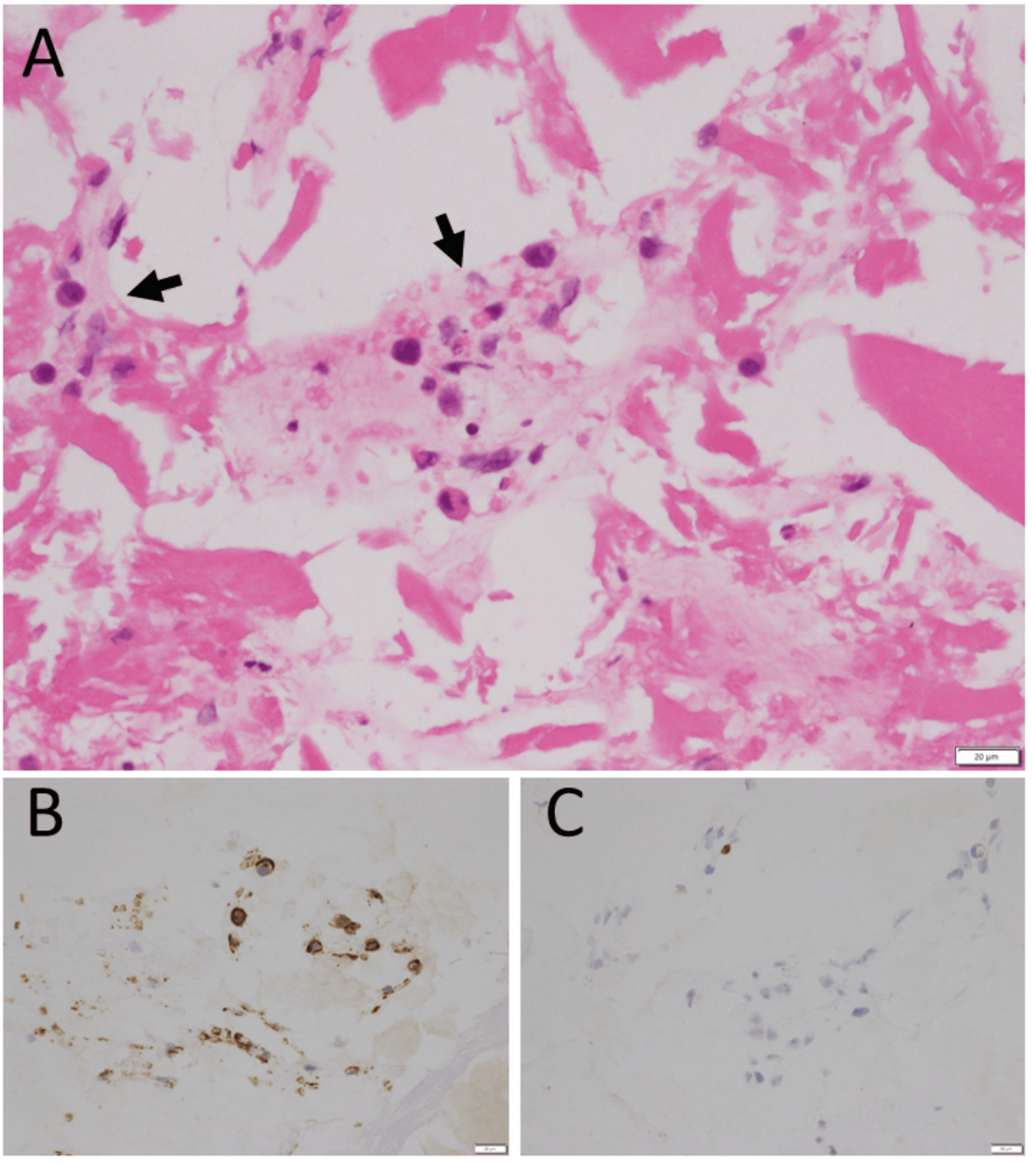
Vitrectomy fluid cytology Vitrectomy fluid cytology from cataract surgery combined with vitrectomy for vitreous opacity in the left eye at age 68. Large anomalous cells (arrows) among lens fragments in hematoxylin-eosin stain (A), positive for CD20 (B) and negative for CD3 (C). Class IV on Papanicolaou classification, indicative of large B cells. Bar: 20 µm

A 68-year-old man came to the first hospital after his family noticed a personality change (e.g., talking less, lack of facial expressions). Magnetic resonance imaging disclosed a mass in the left frontal lobe (Figure 1A-1C), and a brain biopsy via craniotomy confirmed diffuse large B-cell lymphoma (Figure 2A-2E). One year earlier, an ophthalmologist at another hospital had diagnosed him with uveitis in the left eye and treated him with oral prednisolone (20 mg daily), which was unsuccessful, so he underwent cataract surgery combined with vitrectomy in the left eye at the first hospital two weeks before the onset of the personality change. Upon cytological examination, the vitrectomy fluid in the left eye was deemed class IV on the Papanicolaou classification scale (Figure 3A-3C).

The patient received four courses of high-dose methotrexate (3000 mg/m^2^ body surface=4800 mg) and high-dose cytarabine (1500 mg/m^2^ body surface=2400 mg, twice), in combination with rituximab, and also with an intrathecal injection of methotrexate (15 mg), cytarabine (40 mg), and prednisolone (10 mg) in each course over four months, which led to complete remission as confirmed by magnetic resonance imaging. He was followed with no treatment until age 71, when he underwent a cholecystectomy for gallbladder cancer and one course of gemcitabine and cisplatin, based on the cancer's stromal invasion, at a different hospital. He was lost to follow-up at the first hospital and came back four years later at age 75 when he noticed forgetfulness.

**Figure 4:**
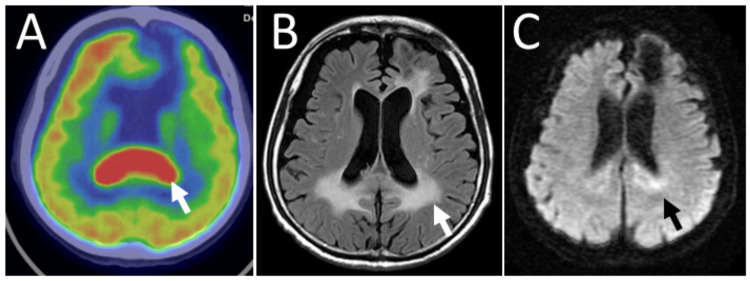
Positron emission tomography and magnetic resonance imaging at age 75 Fluorodeoxyglucose positron emission tomography of the patient at age 75 showing high uptake (arrow) in the splenium of the corpus callosum (A). Magnetic resonance imaging after one course of rituximab combined with high-dose methotrexate showing high signals (arrows) along the corpus callosum on T2-weighted FLAIR image (B) and diffusion-weighted image (C) FLAIR: fluid-attenuated inversion recovery

Fluorodeoxyglucose positron emission tomography (Figure 4A) showed high uptake in the splenium of the corpus callosum, indicating a suspected relapse of brain lymphoma. Another round of brain biopsy was recommended, but the patient refused. Based on the clinical diagnosis of relapse of brain lymphoma, according to magnetic resonance imaging (Figure 4B-4C), he underwent two courses of high-dose methotrexate in combination with rituximab over one month and then two courses of high-dose methotrexate only for one month. Afterwards, he had rituximab monotherapy every month for one year and then every two months for one year, for a total of 17 treatments over two years until age 77, at which point magnetic resonance imaging showed complete remission. At age 76, he also underwent cataract surgery combined with vitrectomy at the first hospital for vitreous opacity in the right eye.

**Figure 5:**
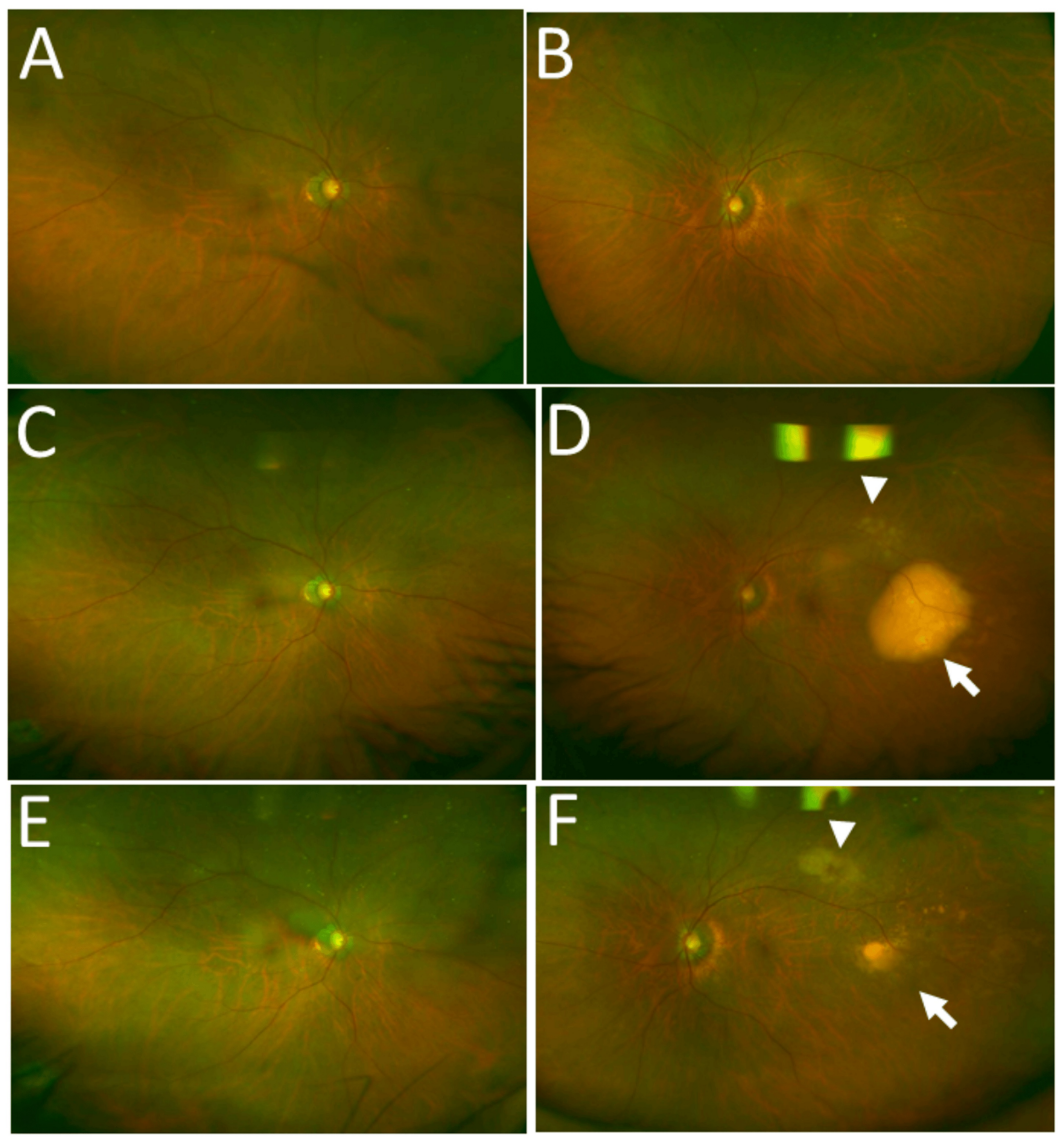
Wide-field fundus photographs at ages 78 and 79 Wide-field fundus photographs of the patient at age 78 showing no lesion in the right eye (A) or the left eye (B). Three months later, the right eye still had no lesion (C), but the left eye exhibited a large yellowish-white subretinal choroidal lesion (arrow) temporal to the macula (D), as well as a smaller lesion upward (arrowhead, D). At age 79, six months after the whole-left-eye radiation (40 Gy), the right eye still had no lesion (E), and scarring lesions (arrow, arrowhead) appeared in the left eye (F)

At age 78, he was referred to an ophthalmologist at our hospital. He was healthy, with no neurological signs or symptoms, and took no medications. The best-corrected visual acuity was 0.7 (decimal scale) in both eyes, with an intraocular pressure of 18 mmHg in the right eye and 15 mmHg in the left eye. Slit-lamp examinations revealed 2+ mutton-fat keratic precipitates with no aqueous cells in both eyes, with intraocular lens implantation. The fundus examinations showed no retinal lesions except for glaucomatous optic disc changes in both eyes (Figure 5A-5B). He was prescribed topical 0.1% betamethasone twice daily and a solution of 0.5% timolol with 0.005% latanoprost once daily.

**Figure 6:**
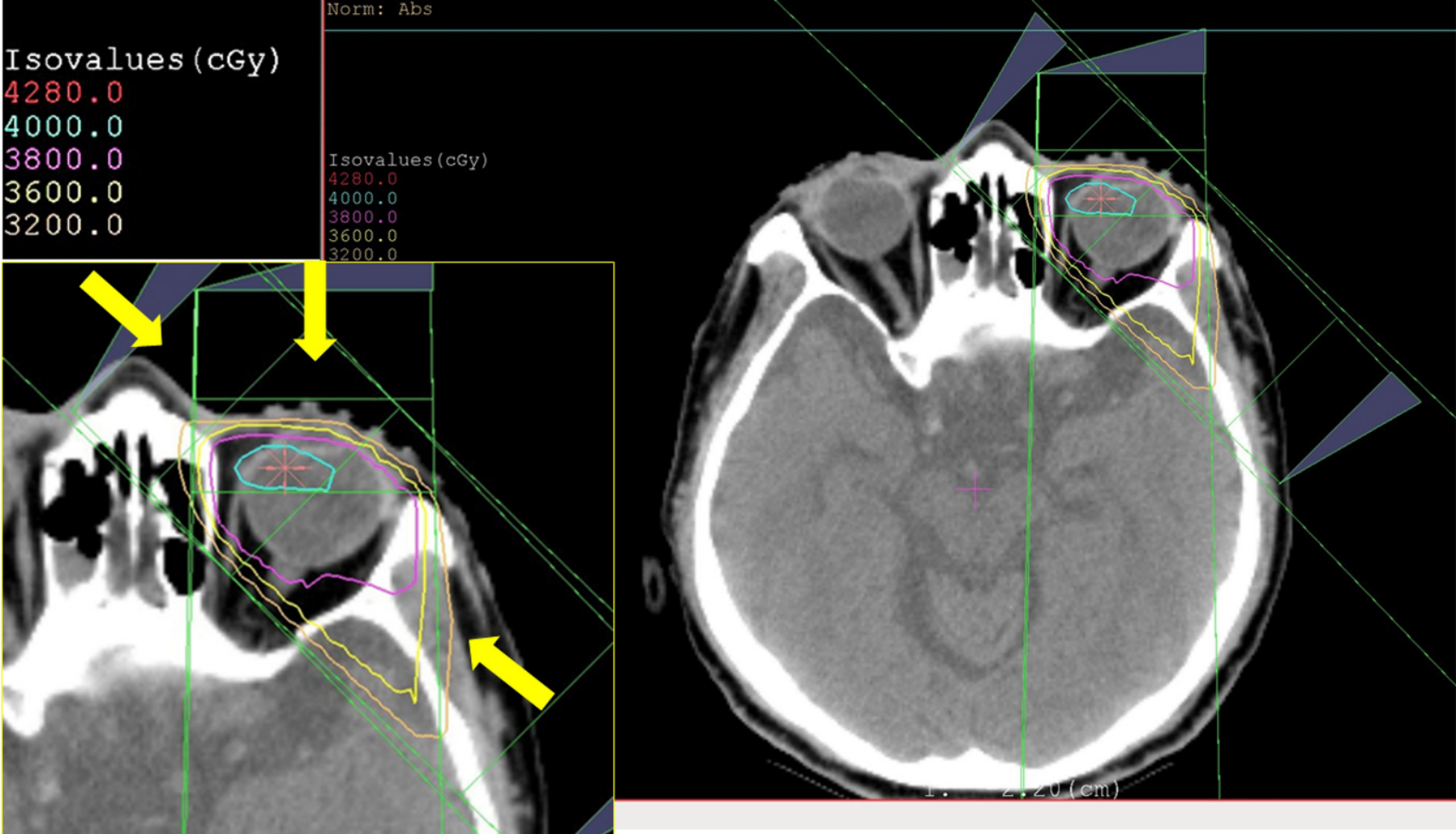
Treatment plan for whole-eye radiation in the left eye Radiation treatment plan for the left eye using three-port irradiation (yellow arrows). The left enlarged inset shows the dose distribution in different colors

Three months later, at follow-up, he complained of blurring in the left eye. The best-corrected visual acuity was 0.7 in the right eye and 0.1 in the left eye, and intraocular pressure was 10 mmHg in both eyes. He had no keratic precipitates, but a large yellowish-white subretinal choroidal mass appeared temporal to the macula, as well as additional small subretinal lesions in the left eye (Figure 5C-5D). He was referred to a hematologist to discuss treatment options, including systemic chemotherapy or local therapy, such as whole-eye radiation. Whole-eye radiation was recommended for the left eye using a total dose of 40 Gy (2 Gy each in 20 fractions). The patient's head was immobilized with the aid of a thermo-plastic shell, and no gaze fixation target was used. The whole eyeball was defined as a clinical target volume (CTV), which was then expanded to a planning target volume (PTV) with a 5-mm auto-isotropic margin (Figure 6). Three-port irradiation (left eye: gantry angle: 0/135/315 degrees) was performed to minimize the irradiation volume in the normal brain. Since the tolerated doses for the eye and optic nerve were considered to be 45 Gy and 50 Gy, respectively, in conventional fractionated radiation at our institution, the eye and optic nerve doses were not measured at the patient's maximum dose of 40.5 Gy. In six months, his left-eye lesions became atrophic (Figure 5E-5F).

**Figure 7:**
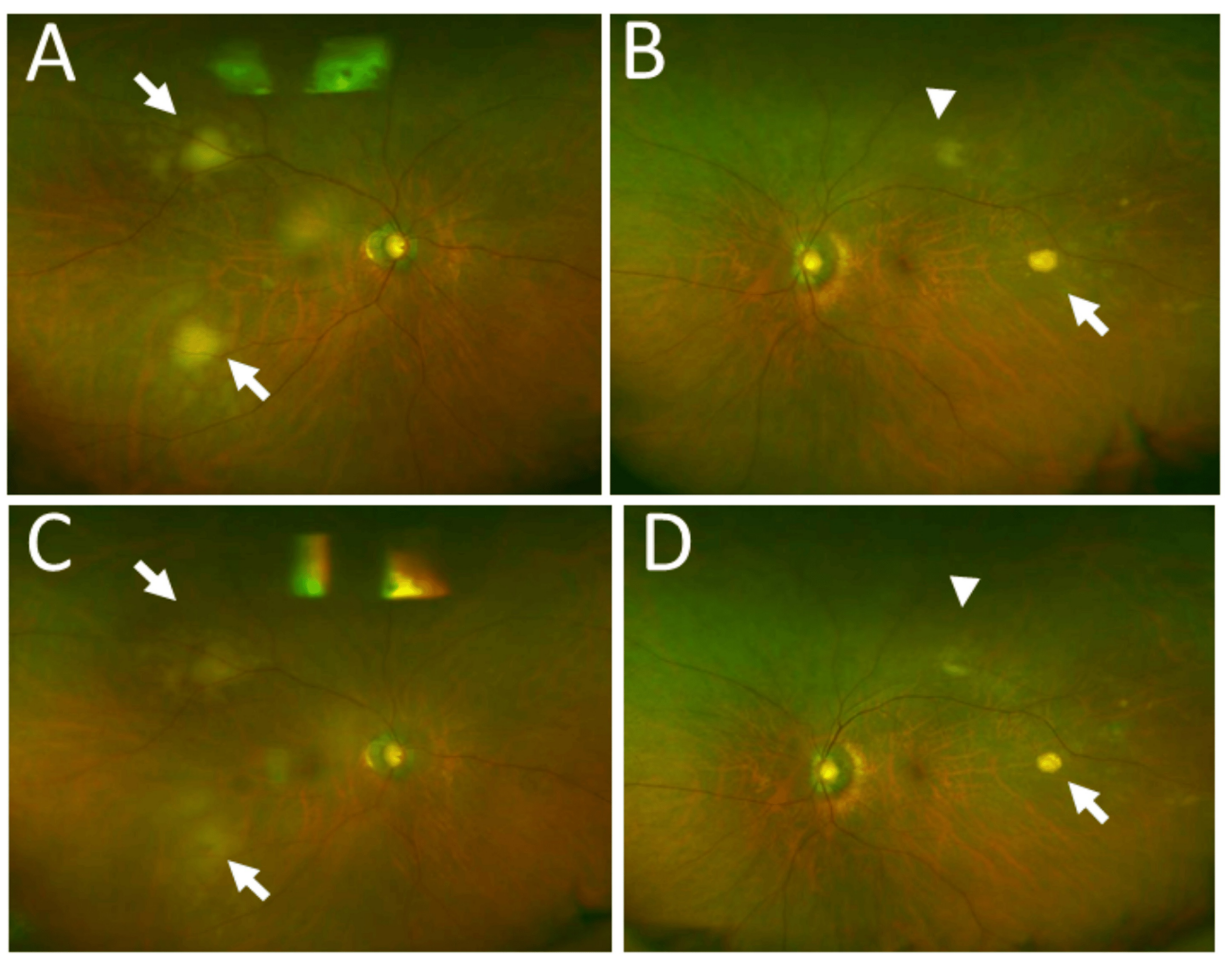
Wide-field fundus photographs at ages 79 and 80 Wide-field fundus photographs of the patient at age 79, one year after the development of left-eye lesion, showing two choroidal lesions (arrows) in the right eye (A) and scarring lesions (arrow, arrowhead) in the left eye (B). Six months after whole-right-eye radiation (40 Gy) at age 80, scarring lesions (arrows, arrowhead) appear in the right eye (C) and the left eye (D)

**Figure 8:**
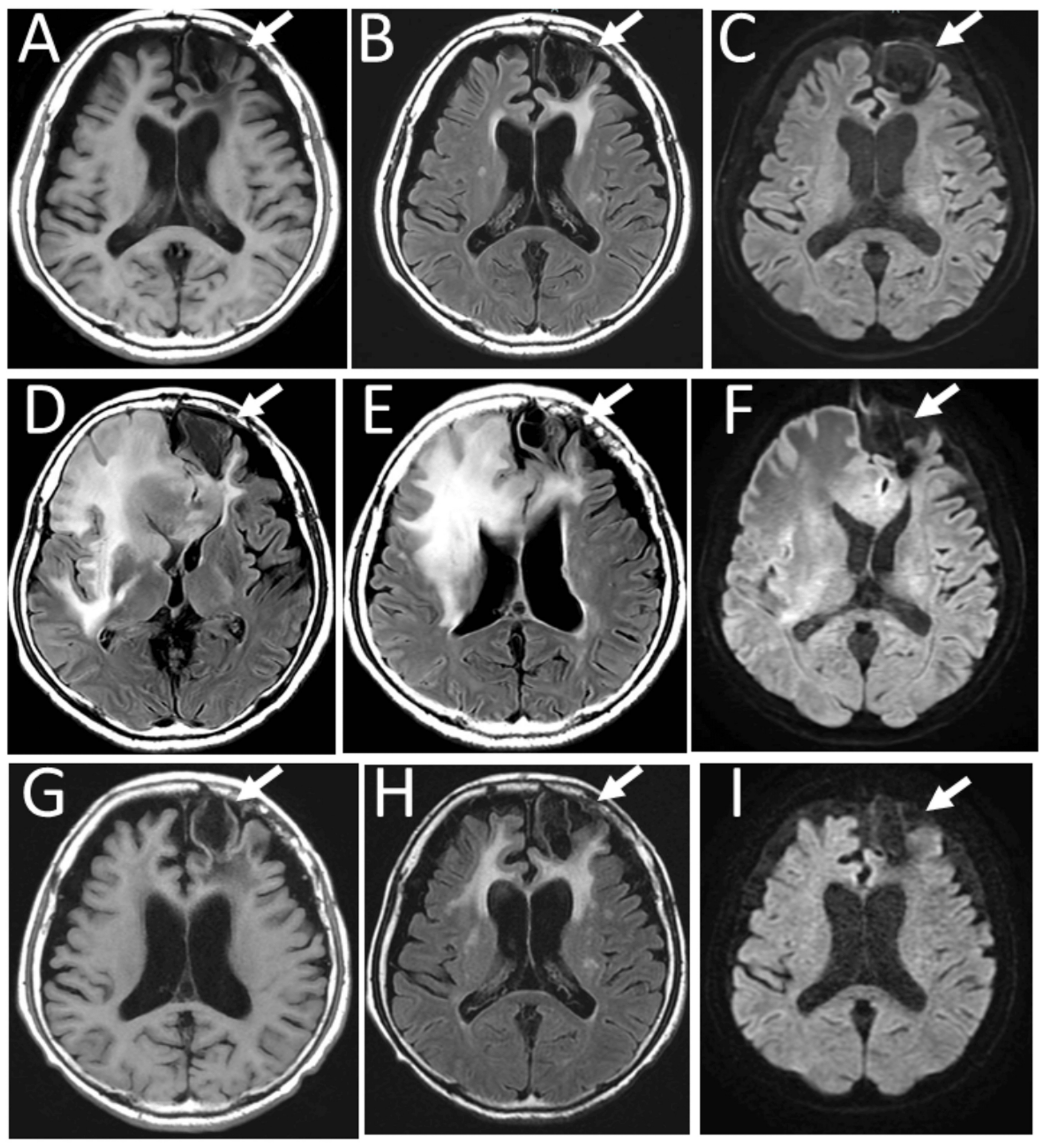
Magnetic resonance imaging at age 81 Magnetic resonance imaging of the patient at age 81 at the annual check-up showing old scarring lesion only in the left frontal lobe (arrows) on T1-weighted image (A), T2-weighted FLAIR image (B), and diffusion-weighted image (C). Five months later, magnetic resonance imaging showed new lesions with high signals from the right frontal to temporal lobes on FLAIR images (D, E) and diffusion-weighted image (F). The new lesions subsided in four weeks with oral tirabrutinib (480 mg daily) as salvage chemotherapy (G: T1-weighted image; H: FLAIR image; I: diffusion-weighted image) FLAIR: fluid-attenuated inversion recovery

At age 79, one year after the onset of the left-eye lesions, he developed two subretinal choroidal lesions in the right eye, temporal to the macular area (Figure 7A-7B). The best-corrected visual acuity was 1.0 in the right eye and 0.6 in the left eye. He underwent whole-eye radiation to the right eye using a total dose of 40 Gy (2 Gy each in 20 fractions) in the same manner as the left eye. The right-eye lesions subsided in six months (Figure 7C-7D). Seven months later, a follow-up magnetic resonance imaging showed no brain relapse (Figure 8A-8C).

**Figure 9:**
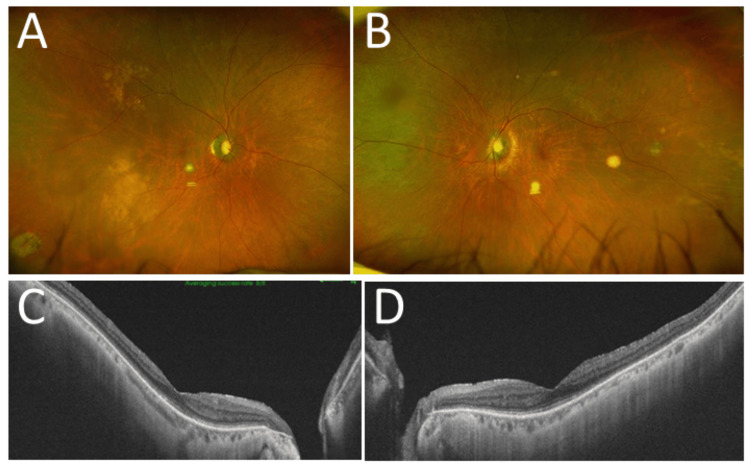
Wide-field fundus photographs and optical coherence tomography at age 81 Images of the patient at age 81, three months after brain relapse, showing stable scarring lesions (A, B) with an almost normal macula in the horizontal images of optical coherence tomography (C, D) in both eyes (A, C: right eye; B, D: left eye)

The patient remained healthy with no systemic medications except topical 0.1% hyaluronan eye drops until age 81, when he abruptly could not move his body and lost bladder control and spatial orientation. Magnetic resonance imaging showed a new, large, high-signal lesion encompassing the right frontal and temporal lobes (Figure 8D-8F), compared with no such lesion six months previously (Figure 8A-8C). Oral tirabrutinib (480 mg daily), a Bruton tyrosine kinase inhibitor, was prescribed as salvage chemotherapy, leading to a conspicuous reduction of the brain lesion in four weeks (Figure 8G-8I). He was stable with mild disorientation at home. At his final ophthalmologist visit three months later for the brain relapse, the best-corrected visual acuity was 0.7 in both eyes, with an intraocular pressure of 12 mmHg in the right eye and 10 mmHg in the left eye. Topical 0.1% hyaluronan was prescribed. Fundus examinations showed no intraocular relapse (Figure 9A-9D).

One month later from the last ophthalmologist visit, he returned by emergency transport to a hematologist with marked disorientation from a brain relapse. He underwent CyberKnife radiotherapy five times in a week for lymphoma lesions in the right parietal lobe and the splenium of the corpus callosum. Oral tirabrutinib was discontinued. He received the best supportive care and died three months later at age 82.

## Discussion

The patient presented to the first hospital at age 68 with primary central nervous system lymphoma and a one-year history of vitreous opacity in the left eye. Primary intraocular lymphoma may have preceded the central nervous system lymphoma by one year. He achieved complete remission with the standard chemotherapy of rituximab combined with high-dose methotrexate and high-dose cytarabine. Seven years later, at age 75, he achieved complete remission for a relapse of brain lymphoma using rituximab combined with high-dose methotrexate. For two years until age 77, he received rituximab monotherapy every month for one year, followed by every two months for one year. During the period in which he received no systemic treatment, he developed choroidal mass lesions in his left eye at age 78 and in his right eye at age 79. No vitreous opacity was found on the occasion of choroidal mass development, which appeared as typical subretinal choroidal lymphoma lesions. The relapse on the opposite side of the brain at age 81 showed a marked response to oral tirabrutinib, a Bruton tyrosine kinase inhibitor, but he died seven months later from the relapse. Throughout the 14-year course, he maintained visual acuity good enough for reading and daily living.

A hematologist was consulted regarding a treatment strategy for the choroidal lymphoma lesions to discuss whether to use systemic chemotherapy or local eye therapy, such as radiation and intravitreal methotrexate injection. As the patient had no relapse in the central nervous system after two rounds of chemotherapy for either the initial lesion or the relapse, whole-eye radiation was recommended and expected to lead to complete remission. Concerns about radiation-induced cataracts were mitigated because the patient had undergone cataract surgery with intraocular lens implantation in both eyes. The radiation oncologist thus administered a total dose of 40 Gy to each eye with curative intent, and the choroidal lesions degenerated in two months.

Radiation-induced mucositis on the ocular surface, including the cornea and conjunctiva, was treated using topical 0.1% hyaluronan eye drops. Other topical eye drops, such as betamethasone and a solution of latanoprost and timolol, were discontinued to avoid irritating the ocular surface. The patient did not develop ocular surface symptoms during the course of radiotherapy and remained symptom-free until his death at age 82. On the other hand, radiation-induced retinopathy should be considered on a yearly basis as a long-term adverse event of whole-eye radiation. The present patient did not develop retinal hemorrhages and cotton-wool spots in 2-3 years after whole-eye radiation in both eyes until the last follow-up.

Radiation therapy has long been part of the standard treatment for Hodgkin lymphoma. In other types of lymphoma, involved-site radiation therapy is a useful curative and palliative treatment contributing to patients' long-term survival [9-12]. Whole-eye radiation also has been reappraised as a curative treatment in primary intraocular lymphoma [13-16]. Vitreous opacity alone, with no retinal or choroidal manifestation in intraocular lymphoma, can be managed with vitrectomy to remove the vitreous opacity, and vitrectomy alone with no additional treatment usually prevents local relapse in the eye [17,18]. However, central nervous system lymphoma can occur, even with prophylactic systemic chemotherapy, after a diagnosis of primary intraocular lymphoma by vitrectomy for vitreous opacity [18,19]. Intraocular lymphoma also might present initially as choroidal lesions or as a local relapse, as in the case presented here. In the situation of intraocular relapse involving either vitreous opacity or choroidal lesions, whole-eye radiation can be an effective treatment to induce local control [4,17,18].

Whole-brain radiation in primary central nervous system lymphoma is a last resort in the standard treatment protocol due to severe side effects, such as unacceptable nausea and deterioration of cognitive function. Because of the old age in the present patient, localized irradiation by CyberKnife radiotherapy was chosen at the last moment, taking into consideration the impact on the quality of life by higher brain dysfunction, which would follow whole-brain radiation. In contrast, whole-eye radiation usually does not cause severe adverse events. Systemic chemotherapy, which treats brain lesions, can also reduce vitreous opacity and choroidal lesions in the setting of concurrent intraocular lymphoma [20]. However, it should be noted that extensive treatment for primary intraocular lymphoma with repeat intravitreal methotrexate injections and prophylactic systemic chemotherapy cannot prevent central nervous system lymphoma from developing later [18,19].

As in this case, primary intraocular lymphoma can manifest as vitreous opacity in both eyes. The patient then relapsed, exhibiting subretinal choroidal lesions in both eyes. Choroidal lesions are difficult to approach by biopsy, in contrast with vitreous opacity, which can be approached safely by vitrectomy and diagnosed pathologically using the cell-block technique on vitrectomy fluid specimens [17-19,21]. The lack of definitive pathological diagnosis of choroidal lesions in both eyes is a significant concern since a treatment decision as whole-eye radiotherapy was based solely on clinical findings. Our rationale would be that the cytological examination of the vitrectomy fluid in the left eye in the early phase of the disease was deemed class IV on the Papanicolaou classification scale. After treating the intraocular lymphoma in both eyes by radiation at a curative dose of 40 Gy, the patient experienced a second relapse of brain lymphoma in the course of two preceding rounds of systemic chemotherapy for the initial lesion and the relapse. This patient's case suggests that the brain lesion and intraocular lesion of primary central nervous system lymphoma behaved independently of each other.

## Conclusions

We describe the case of a patient who developed initial primary central nervous system lymphoma at age 68 and relapsed at age 75. Both the initial lesion and relapse were treated using rituximab combined with high-dose methotrexate-based chemotherapy, which induced complete remission both times. During the period of complete remission in which he received no treatment, he developed choroidal lesions as intraocular lymphoma in the left eye at age 78 and then in the right eye at age 79. Whole-eye radiation was used on both occasions and resulted in the scarring of the choroidal lesions, and he maintained visual acuity good enough for reading and daily living in both eyes.

Primary central nervous system lymphoma and primary intraocular lymphoma might develop independently of each other in the same patient at different times. Systemic chemotherapy for a brain lesion can reduce concurrent intraocular lesions but cannot prevent intraocular lesions from developing later, and prophylactic chemotherapy after the diagnosis of primary intraocular lymphoma similarly cannot prevent brain lesions from developing later. In such cases, whole-eye radiation could be considered as a local treatment to control active intraocular lesions in patients who are not undergoing systemic treatment for primary central nervous system lymphoma. In summary, the finding that brain lesions and intraocular lesions may develop and recur independently has important implications for future treatment strategies. This study is an example that the brain lesion and intraocular lesion of primary central nervous system lymphoma can respond to the treatment independently of each other.
